# Supplementary material for: Transcriptional reprogramming of skeletal muscle stem cells by the niche environment
Source: Nat Commun. 2023 Feb 1;14:535. doi: 10.1038/s41467-023-36265-x (PMC9892560; doi:10.1038/s41467-023-36265-x)

Supplementary Information

Transcriptional reprogramming of skeletal muscle stem cells by the niche environment

Felicia Lazure[1,2,†], Rick Farouni[1,3,†], Korin Sahinyan[1,2], Darren M. Blackburn[1,2], Aldo Hernández-Corchado[1,3], Gabrielle Perron[1,3], Tianyuan Lu[2,4], Adrien Osakwe[4], Jiannis Ragoussis[1,3], Colin Crist[1,2], Theodore J. Perkins[5,6], Arezu Jahani-Asl[7], Hamed S. Najafabadi[1,3,4*] and Vahab D. Soleimani[1,2,*].

[1]*Department of Human Genetics, McGill University, 3640 rue University, Montréal, QC, H3A 0C7, Canada*

[2]*Lady Davis Institute for Medical Research, Jewish General Hospital, 3755 Chemin de la Côte-Sainte-Catherine, Montréal QC, H3T 1E2, Canada*

[3]*McGill Genome Centre, McGill Institute of Molecular Medicine, 740 Dr Penfield Avenue, Montreal, QC H3A 0G1, Canada*

[4]*Quantitative Life Sciences, McGill University, Montreal, Canada*

[5]*Sprott Center for Stem Cell Research, Ottawa Hospital Research Institute, 501 Smyth Road, Ottawa, ON, K1H 8L6, Canada*

[6]*Department of Biochemistry, Microbiology and Immunology, University of Ottawa, Ottawa, ON, K1H 8M5, Canada*

[7]*Department of Cellular and Molecular Medicine and University of Ottawa Brain and Mind Research Institute, University of Ottawa, 451 Smyth Road, Ottawa, ON K1H 8M5*

[*]*Corresponding authors: Vahab D Soleimani, Tel: +1 514 340 8222 ext. 26136 Fax: +1 514 340 7502, vahab.soleimani@mcgill.ca*

Hamed S Najafabadi, Tel: +1 514 398 5308, hamed.najafabadi@mcgill.ca

†*These authors contributed equally*

Table of contents

Supplementary Tables 1-2
Supplementary Figures 1-15

Supplementary Table 1

LFC > 1, s-value < 0.05

	DDN	DDU	DND	DNN	DNU	DUN	NDN	NDU	NND	NNN	NNU	NUD	NUN	UDN	UND	UNN	UNU	UUD	UUN
Down-irrev	35	0	0	0	0	0	93	0	0	0	0	0	0	0	0	0	0	0	0
Down_rev	0	22	0	0	0	0	0	55	0	0	0	0	0	0	0	0	0	0	0
Up_irrev	0	0	0	0	0	0	0	0	0	0	0	0	64	0	0	0	0	0	20
Up_rev	0	0	0	0	0	0	0	0	0	0	0	58	0	0	0	0	0	50	0
No Effect	20	18	1	258	50	1	257	173	625	13371	667	102	110	1	239	637	5	143	58

LFC > 1, s-value < 0.15, LFC batch < 0.05

	DDN	DDU	DND	DNN	DNU	DUN	NDN	NDU	NND	NNN	NNU	NUD	NUN	UDN	UND	UNN	UNU	UUD	UUN
Down-irrev	51	0	0	0	0	0	312	0	0	0	0	0	0	2	0	0	0	0	0
Down_rev	0	68	0	0	0	0	0	241	0	0	0	0	0	0	0	0	0	0	0
Up_irrev	0	0	0	0	0	1	0	0	0	0	0	0	146	0	0	0	0	0	82
Up_rev	0	0	0	0	0	0	0	0	0	0	0	152	0	0	0	0	0	225	0
No Effect	0	0	1	258	50	0	0	0	625	13371	667	0	0	0	239	637	5	0	0

Supplementary Table 1: Number of genes affected by age, by engraftment, and by the niche, at different thresholds.

The column headers from left to right represent the effect of engraftment, the effect of age and the effect of the niche. N represents no significant effect, U represents upregulation, D represents downregulation. For example, DDN represents downregulated in engraftment, downregulated in age and no significant change in the niche while UDN represents upregulated in engraftment, downregulated in age and no significant change in the niche.

Supplementary Table 2

		Groups of genes of interest							
		All groups	Downregulated			Upregulated			
			All downregulated	irreversible	reversible	All upregulated	irreversible	reversible	
Genes associated to DMRs	Gain	0.098<br>1.2585<br>44	0.6845<br>0.9071<br>17	0.7207<br>0.8552<br>9	0.5773<br>0.9774<br>8	0.0172<br>1.6256<br>27	0.4102<br>1.1466<br>8	0.0091<br>1.9387<br>19	p-value<br>odds ratio<br># of overlapping genes
	Loss	0.2262<br>1.1495<br>42	0.8703<br>0.7651<br>15	0.6363<br>0.9277<br>10	0.9305<br>0.5728<br>5	0.0243<br>1.5711<br>27	0.1903<br>1.4214<br>10	0.0445<br>1.6471<br>17	

Supplementary Table 2: Overlap between DMRs with genes upregulated and downregulated with age

Number of DMRs overlapping with genes that are downregulated and upregulated in aging and reversible and irreversible by the niche.

Supplementary Table 3

		DESeq2 diff. peaks padj < 0.1 and LFC threshold 0.5							
		Groups of genes of interest							
		All groups	Downregulated			Upregulated			
			All downregulated	irreversible	reversible	All upregulated	irreversible	reversible	
Genes associated to ATAC-seq peaks	Upregulated peaks	0.002215<br>1.243<br>274	0.9567<br>0.8508<br>140	0.8402<br>0.8856<br>78	0.9305<br>0.8173<br>62	5.47E-12<br>2.2598<br>134	4.05E-07<br>2.3613<br>65	2.63E-06<br>2.1263<br>69	p-value<br>odds ratio<br># of overlapping genes
	Downregulated peaks	6.01E-08<br>1.476<br>319	4.37E-10<br>1.7093<br>234	0.000136291<br>1.5437<br>120	4.38E-07<br>1.8637<br>114	0.402806702<br>1.0395<br>85	0.732651158<br>0.902<br>36	0.20238199<br>1.1695<br>49	

Supplementary Table 3: Overlap between ATAC-Seq peaks with genes upregulated and downregulated with age

Number of ATAC-seq peaks overlapping with genes that are downregulated and upregulated in aging and reversible and irreversible by the niche.

Supplementary Figure 1

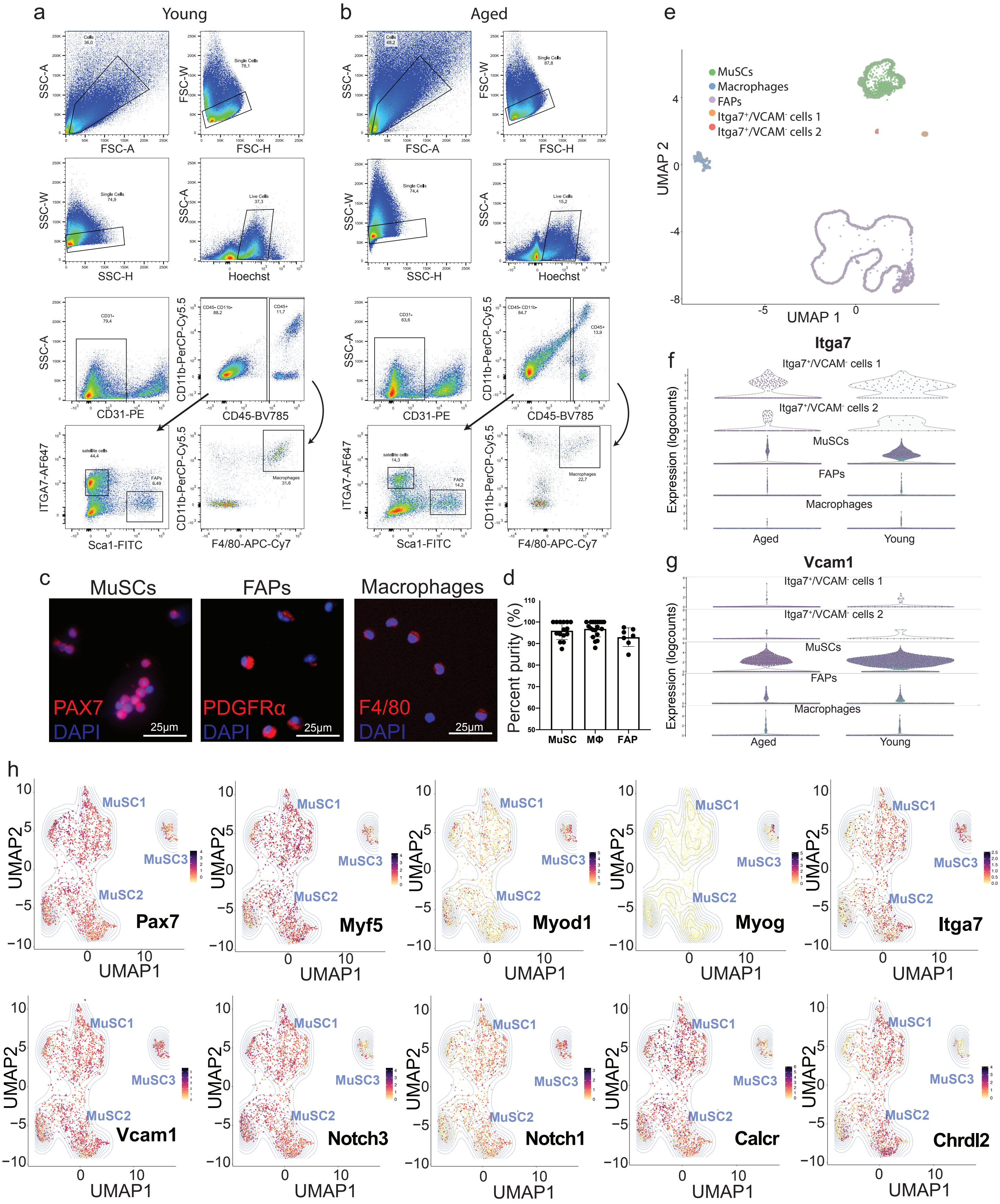

Supplementary Fig. 1: Fluorescence Activated Cell Sorting (FACS) strategy for the simultaneous isolation of pure populations of MuSCs, Fibro-Adipogenic Progenitor (FAPs) cells and muscle-resident macrophages

a-b, Complete FACS sorting strategy for the simultaneous isolation of MuSCs (ITGA7$^+$, CD31$^-$, SCA1$^-$, CD45$^-$, CD11b-)[1], macrophages (F4/80$^+$, CD45$^+$, CD11b$^+$, CD31$^-$)[2], and FAPs (SCA1$^+$, ITGA7$^-$, CD45$^-$, CD11b$^-$, CD31$^-$)[3] from young (a) and aged (b) mice.

c, Immunofluorescent staining of freshly sorted MuSCs, macrophages and FAPs with selected representative markers (PAX7, PDGFRA, F4/80, respectively) of each population. (Scale bar = 25µm)

d, Quantification of the percentage of pure freshly isolated cells based on immunofluorescence of PAX7 in MuSCs (n=1 isolation, n=15 fields of view, n=440 cells counted), PDGFRA in FAPs (n=2 isolations, n=7 fields of view, n=820 cells counted) and F4/80 in macrophages (n=2 isolations, n=19 fields of view, n=656 cells counted). Data are presented as mean values +/- SD.

e, UMAP plot of young and aged MuSC, FAP and macrophage scRNA-seq samples before filtering out two ITGA7$^+$/VCAM1$^-$ cell populations.

f-g, Violin plots showing the expression of Itga7 (f) and Vcam1 (g) in young and aged MuSC, FAP and macrophage scRNA-seq samples before filtering out two ITGA7$^+$/VCAM1$^-$ cell populations.

h, Gene expression plots in the UMAP embedding for muscle-specific genes (*Pax7, Myf5, Myod1*, *Myog*, *Itga7, Vcam1*), and quiescence markers (*Notch3, Notch1, Calcr, Chrdl2*) in young and aged MuSCs.

Supplementary Figure 2

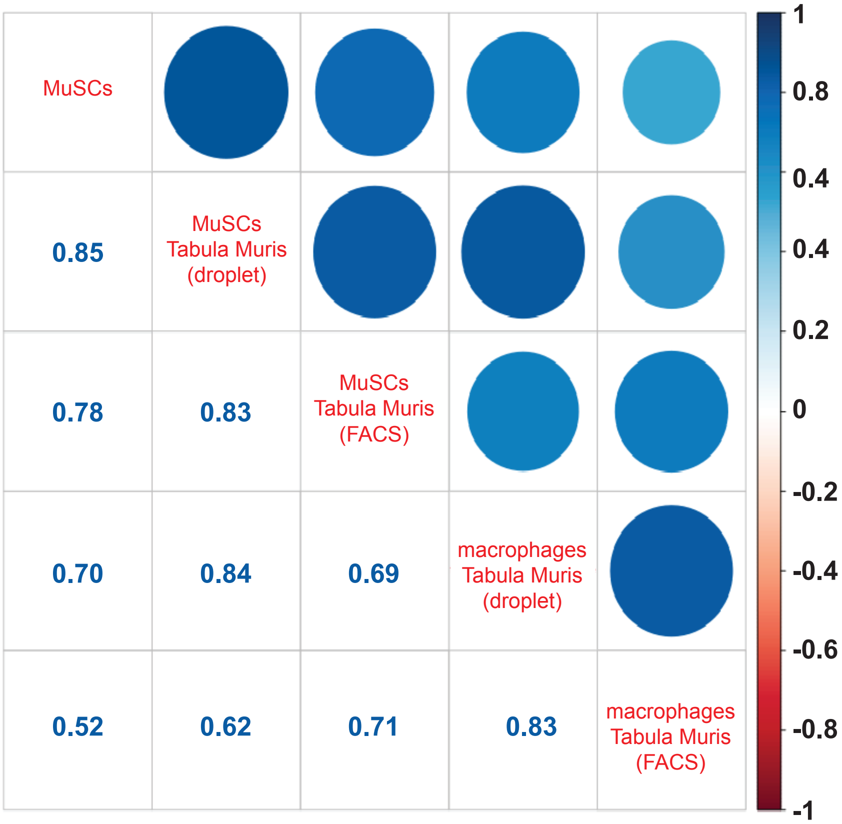

Supplementary Fig. 2: Comparison of MuSC scRNA-Seq data with the Tabula Muris Dataset
Correlation plot showing the concordance between our scRNA-Seq MuSC data and the Tabula Muris[4] MuSC datasets by FACS and droplet methods. Comparison with Tabula Muris macrophages was also included as a negative control.

Supplementary Figure 3

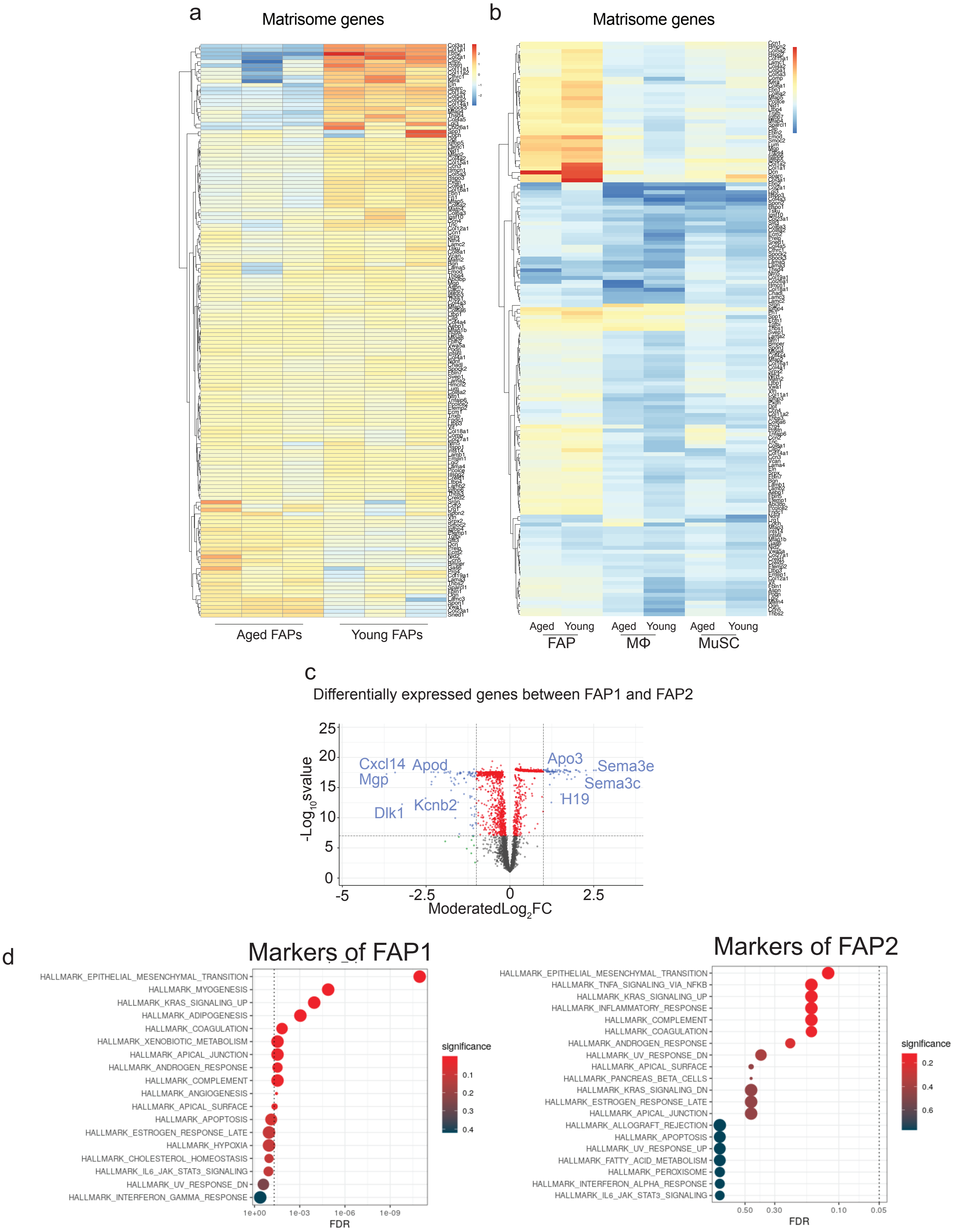

Supplementary Fig. 3: Genes involved in processes such as cytokine signaling and extracellular matrix composition are perturbed in aged MuSCs and niche cells
a, Heat map of matrisome genes in young and aged FAPs (color scale denotes beta-coefficients.
b, Heat map of matrisome genes [5] in young and aged macrophages, FAPs, and MuSCs. (Color scale represents beta-coefficients)
c, Volcano plot of moderated LFC and s-value of the contract between FAP2 and FAP1 clusters. Positive values show genes upregulated in FAP2 subpopulation.
d, Gene set enrichment analysis of positive markers of FAP1 and FAP2 subpopulations using Hallmark gene sets [6].

Supplementary Figure 4

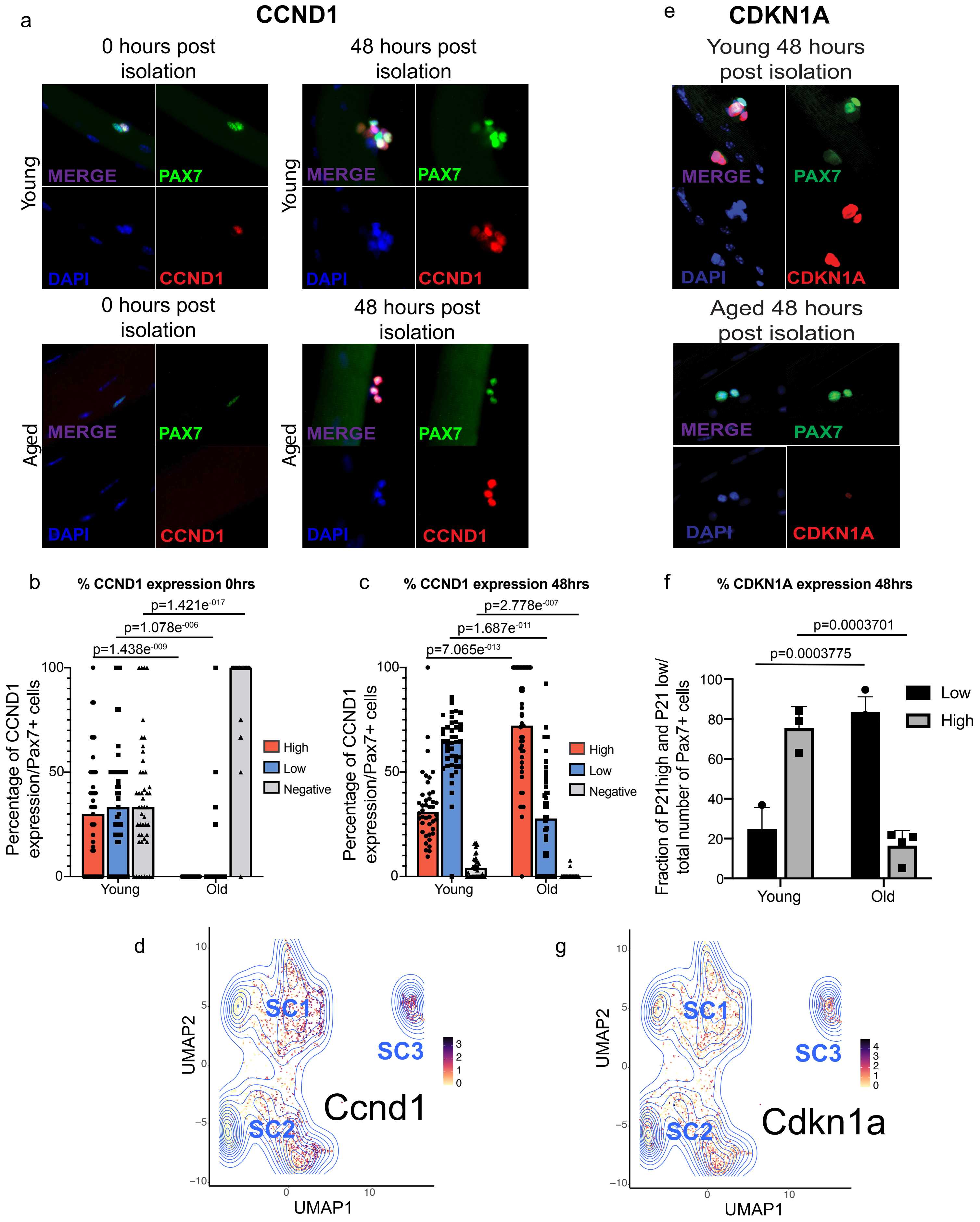

Supplementary figure 4: Aged MuSCs show deregulated cell cycle both at the gene expression and protein level

a, Immunofluorescence of isolated EDL myofibers from 4-week-old and 26-month-old mice for CCND1 and Pax7 at 0h and 48h post isolation.

b, Quantification of high, low, and negative CCND1 expression in Pax7+ MuSCs from young and aged EDL myofibers at 0hr showing heterogeneity in protein expression in young MuSCs (n=3 mice per group, n=42 fibers from young mice, n=39 fibers from aged mice, 2-tailed t-test). Data are presented as mean values +/- SD. $p=1.438e^{-009}$ for high CCND1 expression, $p=1.078e^{-006}$ for low CCND1 expression, $p=1.421e^{-017}$ for negative expression.

c, Quantification of high, low, and negative CCND1 expression in Pax7+ MuSCs from young and aged EDL myofibers after 48h in culture (n=3 mice per group, n=42 fibers from young mice, n=45 fibers from aged mice, 2-tailed t-test). Data are presented as mean values +/- SD. $p=7.065e^{-013}$ for high CCND1 expression, $p=1.687e^{-011}$ for low CCND1 expression, $p=2.778e^{-007}$ for negative expression.

d, Ccnd1 gene expression in the scRNA-Seq UMAP embedding of young and aged MuSCs.

e, Immunofluorescence of isolated EDL myofibers from 4-week-old and 20-month-old mice, cultured for 48 hours and stained for P21 (1:500, Abcam # ab188224) and Pax7.

f, Quantification of high and low P21 expression in Pax7+ MuSCs from young and aged EDL myofibers. (n=3 mice per group, n=53 fibers from young mice, n=62 fibers from aged mice, 2-tailed t-test). Data are presented as mean values +/- SD. p=0.0003775 for low p21 expression, p=0.0003701 for high p21 expression.

g, Cdkn1a gene expression in the scRNA-Seq UMAP embedding of young and aged MuSCs. (n=3 mice per group).

Supplementary Figure 5

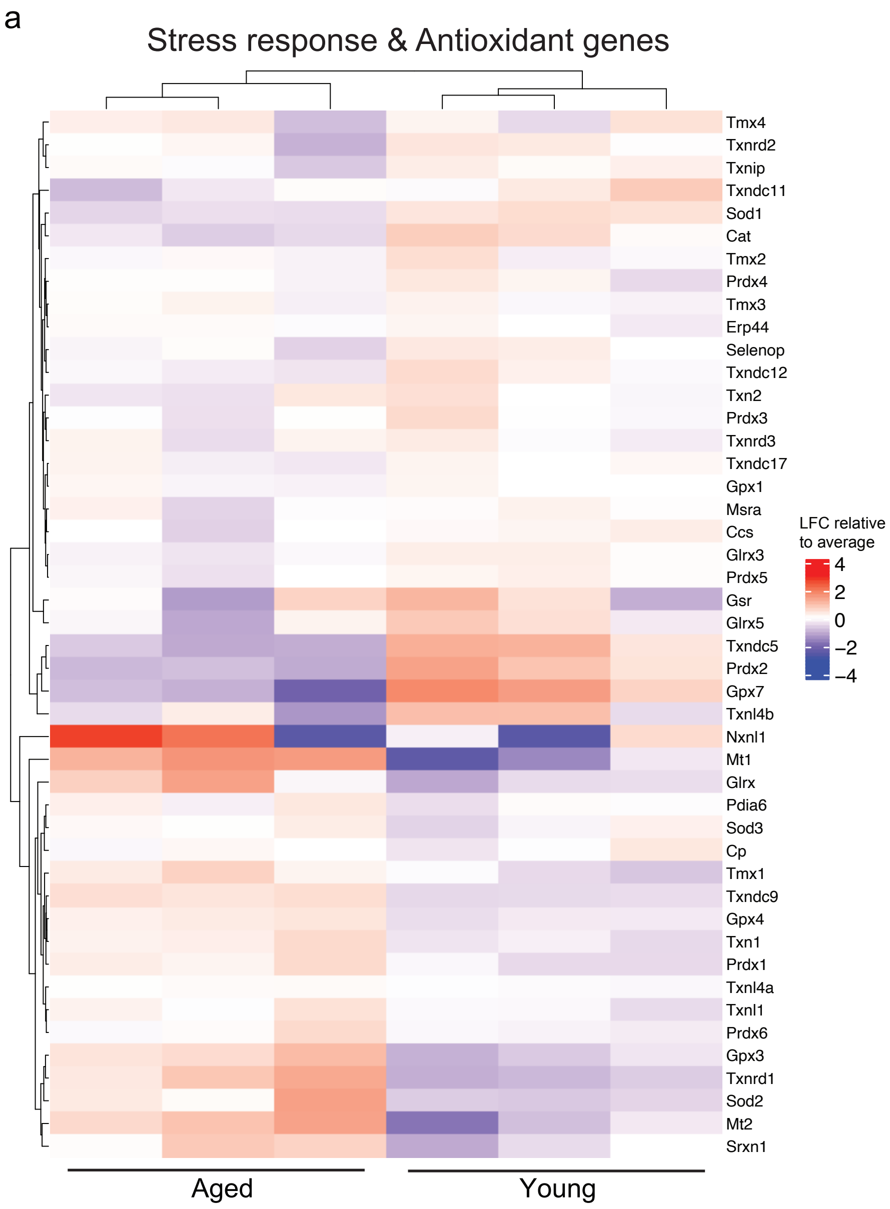

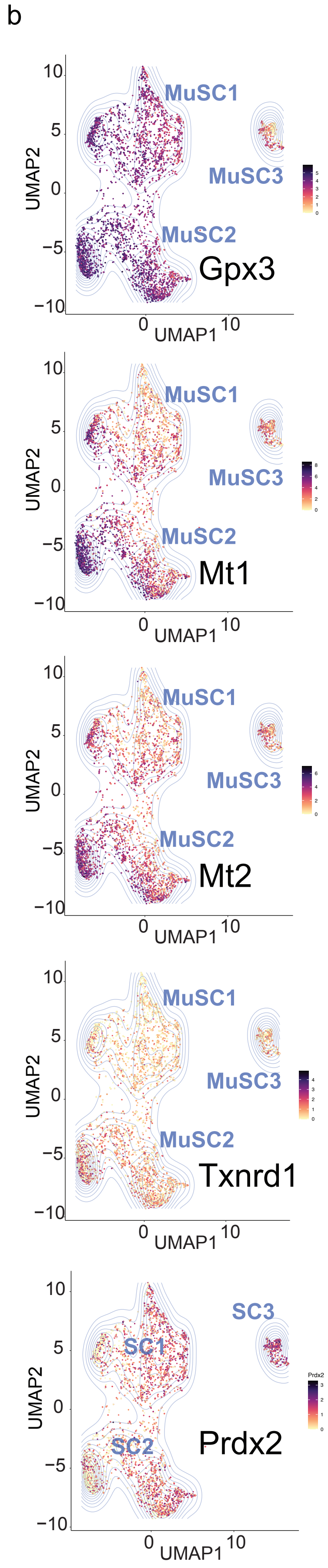

Supplementary Fig. 5: Aged MuSCs display an altered expression profile of stress response genes

a, Heat map showing the LFC relative to the average expression of stress-response genes [7] in young compared to aged MuSCs.

b, Gene expression plots in the UMAP embedding for select stress-response genes that are differentially expressed between young and aged MuSCs.

Supplemental Figure 6

a

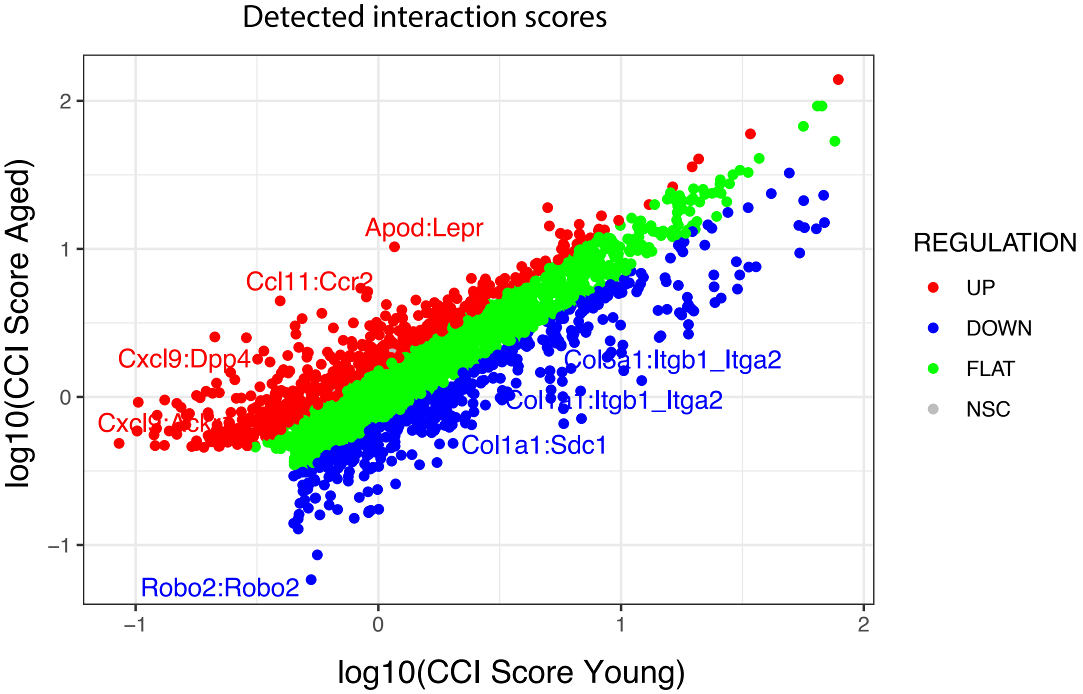

b

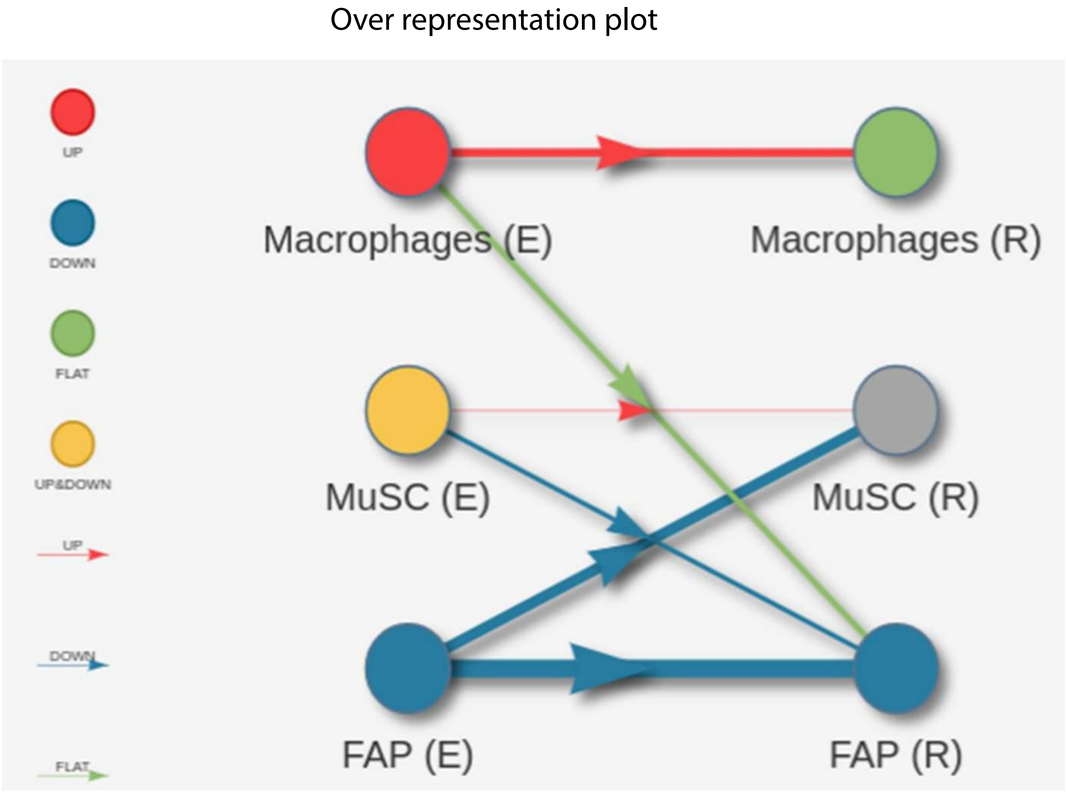

Supplementary Fig. 6: Differential analysis of cell-cell interactions
Results for differential analysis of cell-cell interactions (CCI) using the scDiffCom package. Labels are based on aged samples relative to young samples.
a, Plot of the log-transformed CCI scores for aged and young samples for all detected interactions. Interactions with absolute log-fold changes greater than 2 are labelled.
b, Over-representation network demonstrating which Emitters (E), Receivers (R), and Emitter-Receiver pairs are over-represented in a specific condition.

Supplemental Figure 7

a

Biological Replicate	# of MuSCs Transplanted	# of MuSCs Reisolated	Percentage (%) Reisolated
Young 1	20,000	75	0.375
Young 2	20,000	20	0.10
Young 3	15,000	150	1.00
Young 4	20,000	73	0.365
Young 5	20,000	104	0.520
Aged 1	20,000	44	0.220
Aged 2	20,000	66	0.330
Aged 3	10,000	101	1.01
Aged 4	20,000	33	0.165
Aged 5	20,000	35	0.175

b

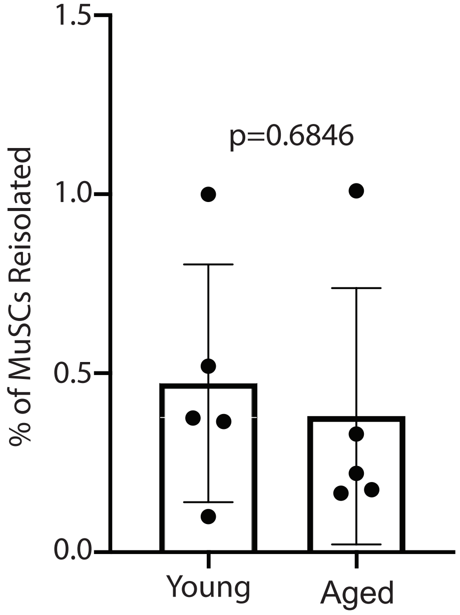

Supplementary Fig. 7: Age effect on donor MuSCs transplantation

a, Table of number and percentage of engrafted donor cells from young and aged in five biological replicates.

b, Quantification of the percentage of reisolated MuSCs post-transplantation (# of MuSCs isolated 21 days post-transplantation / # of MuSCs initially transplanted) (n=5 young and aged mice, two-tailed t-test) showing no statistically significant age effect of donor cells on engraftment efficiency. Data are presented as mean values +/- SD.

Supplementary Figure 8

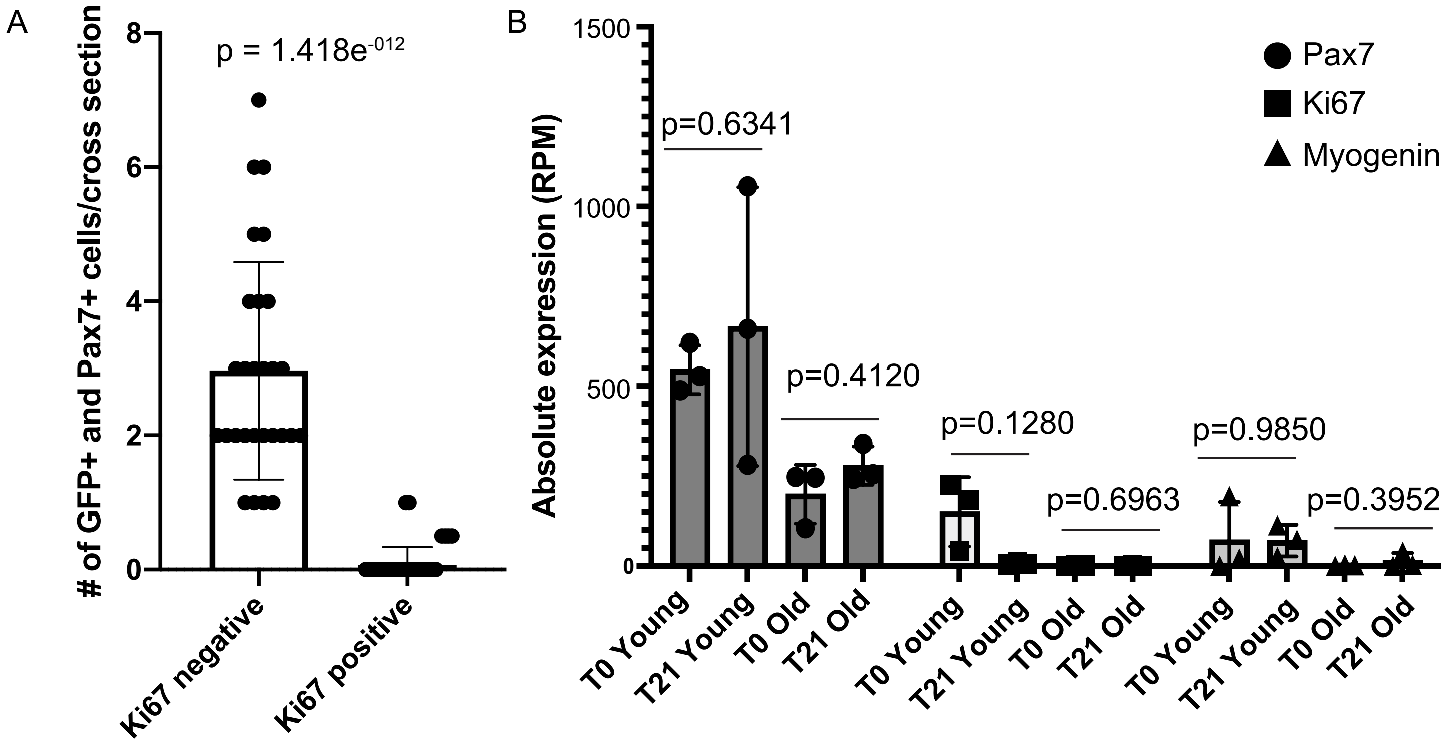

Supplementary Fig. 8: Engrafted MuSCs are quiescent 21 days post-engraftment
a, Quantification of the number of KI67+ and KI67- donor MuSCs per TA cross-section in transplanted host mice (related to Figure 4E) (n=82 donor cells from n=3 mice). Data are presented as mean values +/- SD. $p=1.418e^{-012}$.
b, Gene expression levels (RPM) of *ki67*, *Pax7* and *Myog* in young and aged MuSCs before and after transplantation into young hosts (n=3 mice per group). Data are presented as mean values +/- SD.

Supplementary Figure 9

a

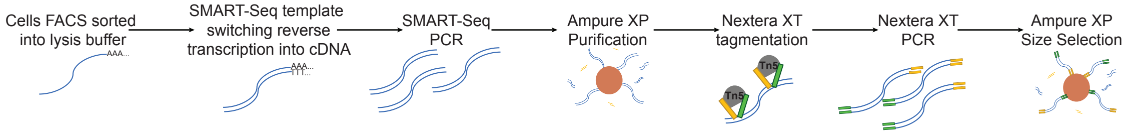

b

Number of cells	Number of PCR cycles used with SMART-Seq
30-50	17
50-100	16
100-150	15
150-400	14
400-1000	12-13
1000	11

c

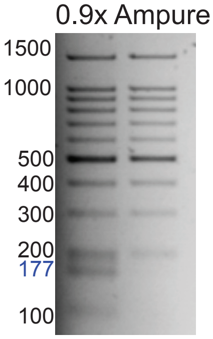

d

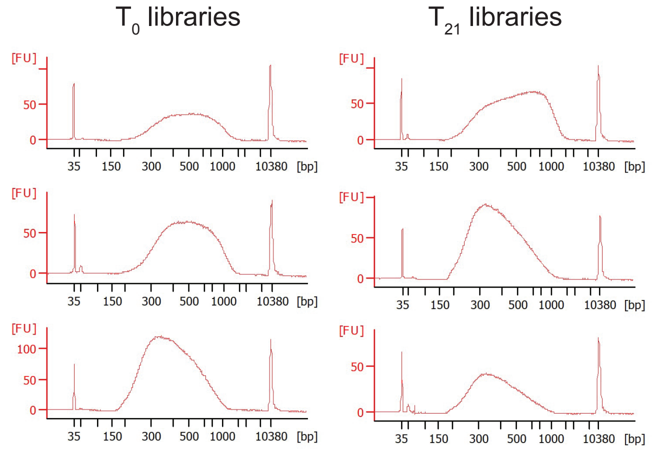

e

	Number of cells	Number of reads	Number of aligned reads	Number of unique reads	Percent aligned reads	Percent unique reads
Aged T0-1	1000	58870695	32673727	11446097	55.5	19.4
Aged T0-2	1000	49688879	28086575	9897017	56.5	19.9
Young T0-1	1000	42920426	26667590	9298834	62.1	21.7
Young T0-2	1000	51092371	33301402	11165875	65.2	21.9
Young T0-3	1000	58770931	39423797	13844857	67.1	23.6
Aged T21-1	38	47596086	31201999	10665092	65.6	22.4
Aged T21-2	171	52524544	35119392	11935463	66.9	22.7
Young T21-1	187	48128644	21102721	8144666	43.8	16.9
Young T21-2	188	56280876	31295112	11329468	55.6	20.1

f

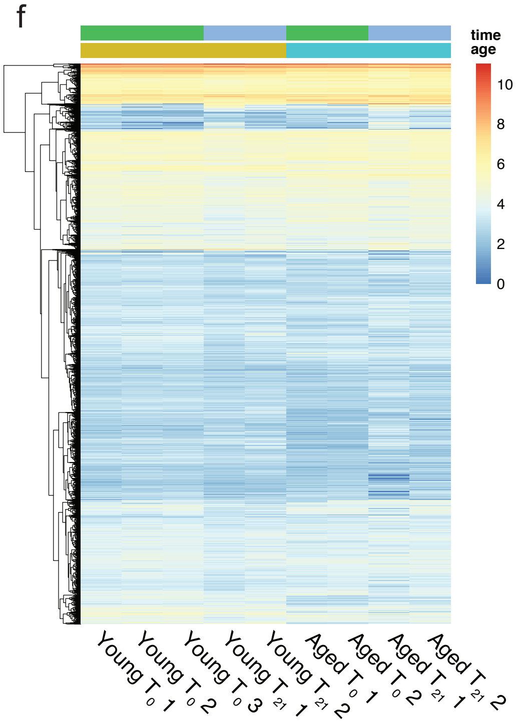

g

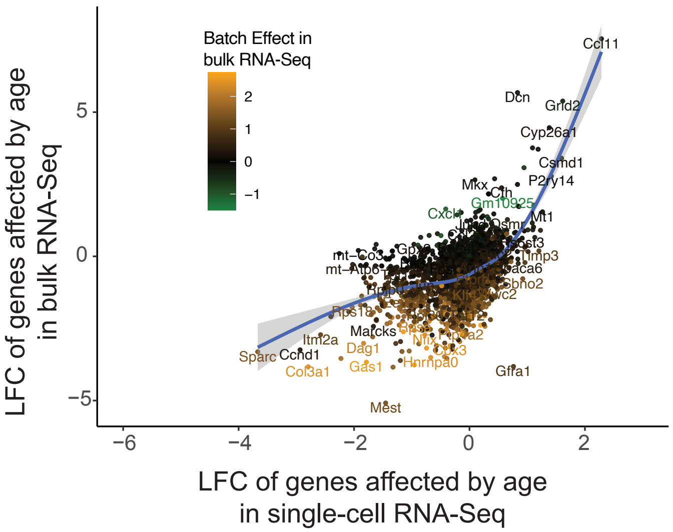

Supplementary Fig. 9: SMART-seq technology allows the generation of RNA-sequencing libraries from rare cell types

a, Schematic diagram of library preparation method using SMART-seq[8] and Nextera XT[9]. Briefly, MuSCs are sorted using FACS directly into SMART-seq reaction buffer, followed by template switching reverse transcription and PCR. After Ampure XP size selection at a 1:1 (v:v) ratio, cDNA is quantified and 0.15ng is used for Nextera XT tagmentation. After PCR addition of Illumina sequencing adapters, libraries are once again purified using Ampure XP at a 1:0.9 (v:v) ratio and sequenced.

b, Number of PCR cycles used with SMART-seq depending on the number of cells sorted by FACS.

c, Representative photo of Ampure XP size selection at a 0.9x ratio on a DNA ladder, visualized on an agarose gel, demonstrating its utility at removing primer dimers of <200bp. (size selection was performed once on a DNA ladder and later validated on n=21 samples by bioanalyzer as shown in d).

d, Example of bioanalyzer profiles of SMART-seq libraries created from 50-1000 cells (size selection was performed independently on n=21 total samples).

e, Table of sequencing read information for SMART-seq libraries generated from different numbers of cells.

f, Heat map of the top 6000 expressed genes in young and aged MuSC samples, at T_0 and at T_{21}, showing consistency within replicates.

g, Scatter plot of the LFCs of the effect of age from bulk RNA-seq and from scRNA-seq showing high concordance of differentially expressed genes from both modalities. Coloring is based on the size of the LFC effect of the allogeneic niche in bulk RNA-seq. Grey area represents the 95% confidence interval for the mean.

Supplementary Figure 10

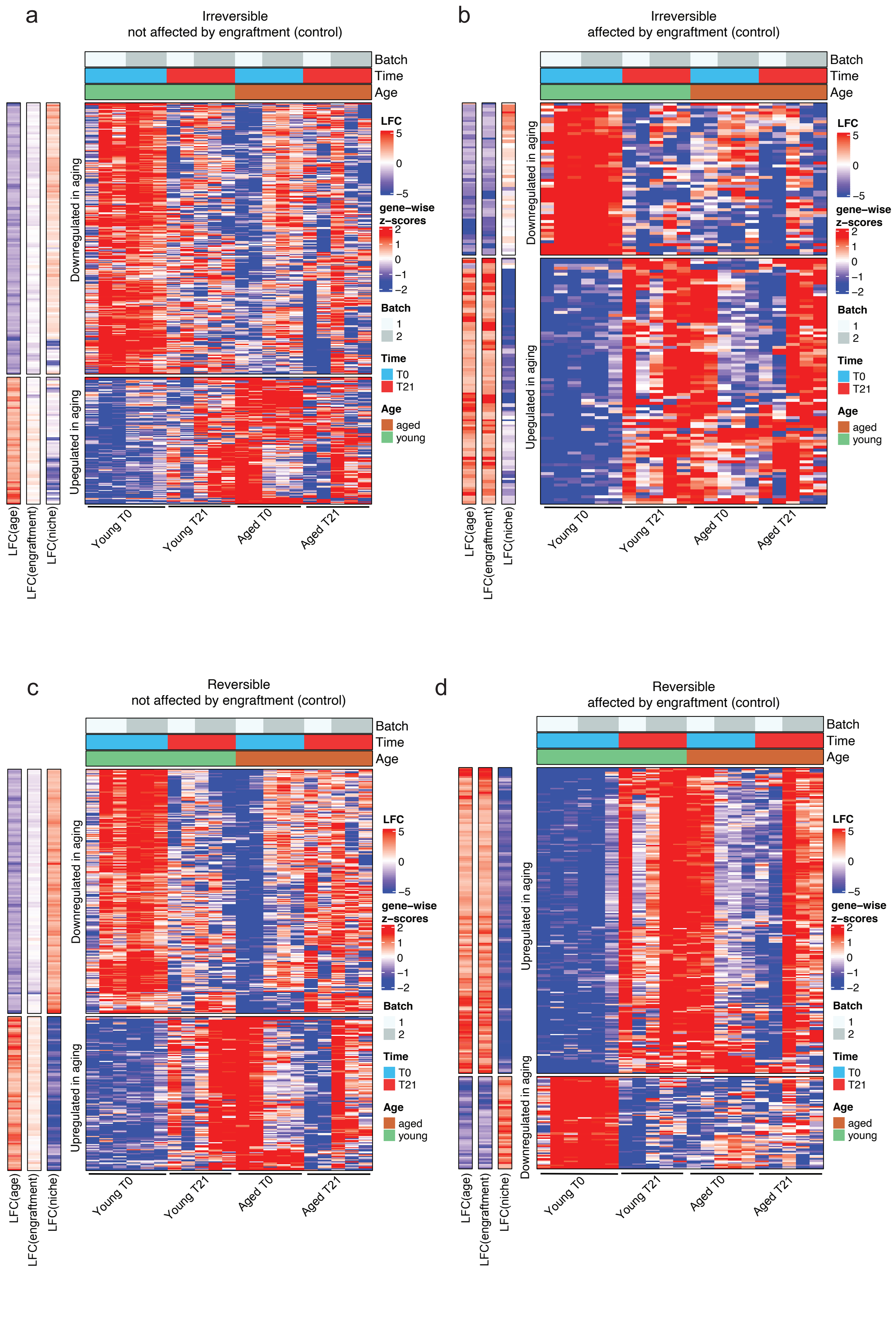

Supplementary Fig. 10: **MuSCs are affected by age, engraftment, and the heterochronic niche after transplantation into a young host**

a, Heat map of the LFC due to engraftment, age, and the niche (left) and z-scores of irreversible age-altered genes that are not affected by engraftment (control).

b, Heat map of the LFC due to engraftment, age, and the niche (left) and z-scores of irreversible age-altered genes that are affected by engraftment (control).

c, Heat map of the LFC due to engraftment, age, and the niche (left) and z-scores of reversible age-altered genes that are not affected by engraftment (control).

d, Heat map of the LFC due to engraftment, age, and the niche (left) and z-scores of reversible age-altered genes that are affected by engraftment (control).

Supplementary Figure 11

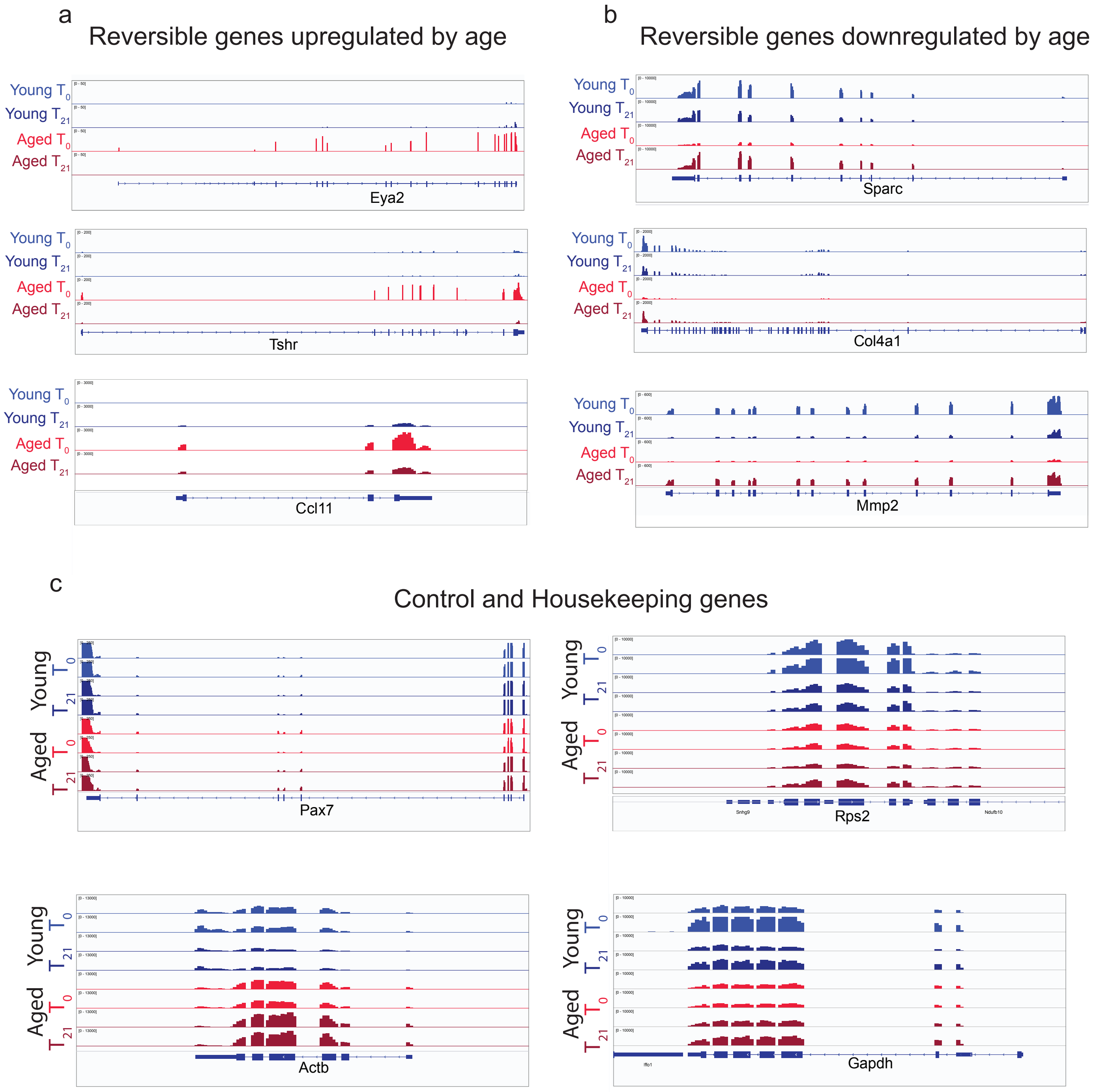

Supplementary Fig. 11: A number of genes in aged MuSCs can be restored to a youthful condition after transplantation into a young niche

a-b, Representative IGV tracks of selected age-upregulated (a) or downregulated (b) genes (*Sparc, Col4a1, Mmp2, Eya2, Gas1, Prdx2, Tshr*) that are reversible to a youthful state by exposure to the young niche in aged MuSCs.

c, Representative IGV tracks of select control and housekeeping genes (*Pax7, Gapdh, Actb, Rps2*) in young and aged MuSCs from SMART-seq analysis at T_0 and T_{21}.

Supplementary Figure 12

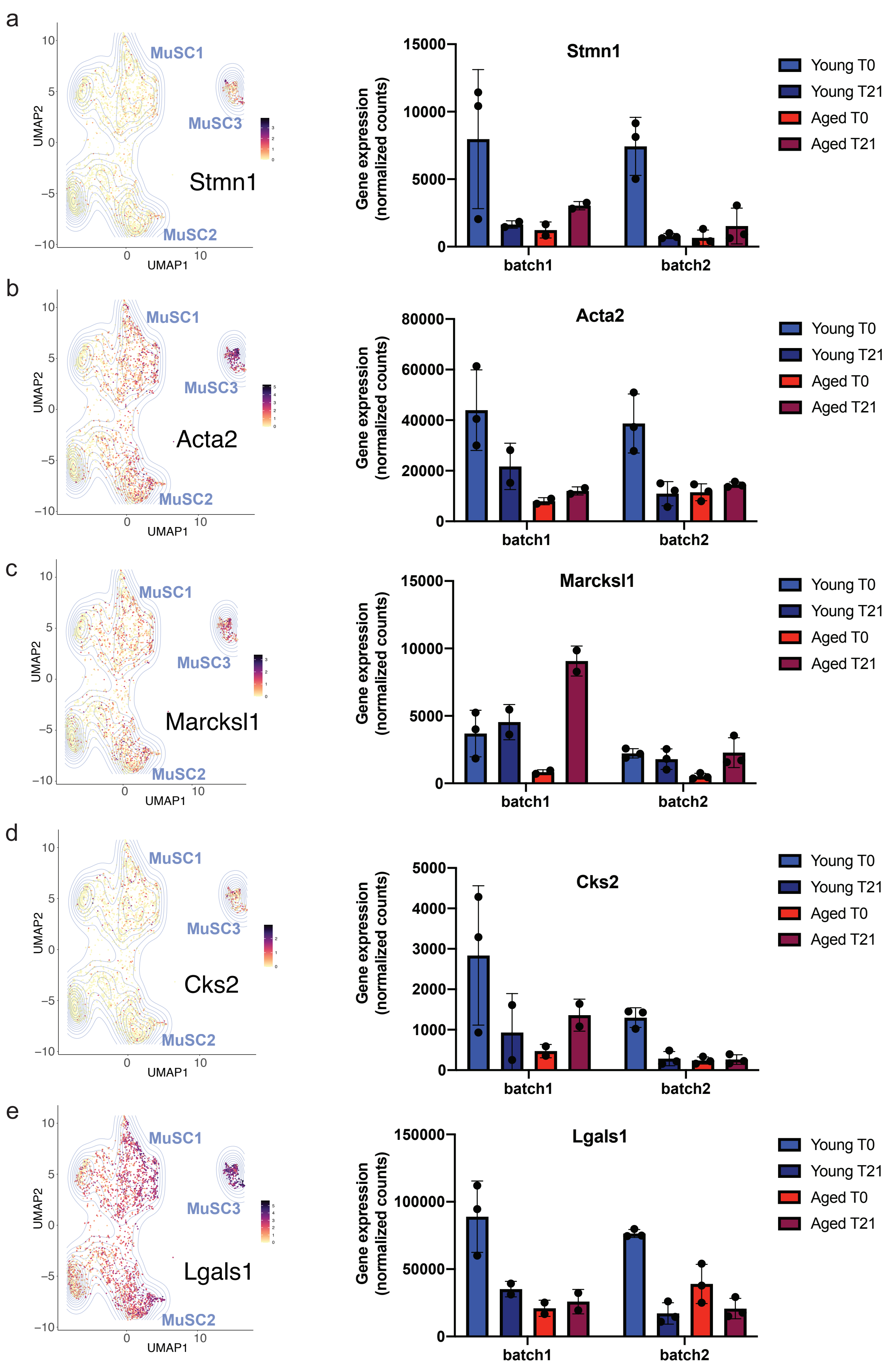

Supplementary Fig. 12: A portion of markers of MuSC Cluster 3 are partially restored in aged MuSCs after engraftment into a young niche

a, UMAP (left) and bar plots representing the absolute expression (right) of *Stmn1*.
b, UMAP (left) and bar plots representing the absolute expression (right) of *Acta2*.
c, UMAP (left) and bar plots representing the absolute expression (right) of *Marcksl1*.
d, UMAP (left) and bar plots representing the absolute expression (right) of *Cks2*.
e, UMAP (left) and bar plots representing the absolute expression (right) of *Lgals1*.
(batch 1: n=2-3 mice per group, batch 2: n=3 mice per group). Data are presented as mean values +/- SD.

Supplementary Figure 13

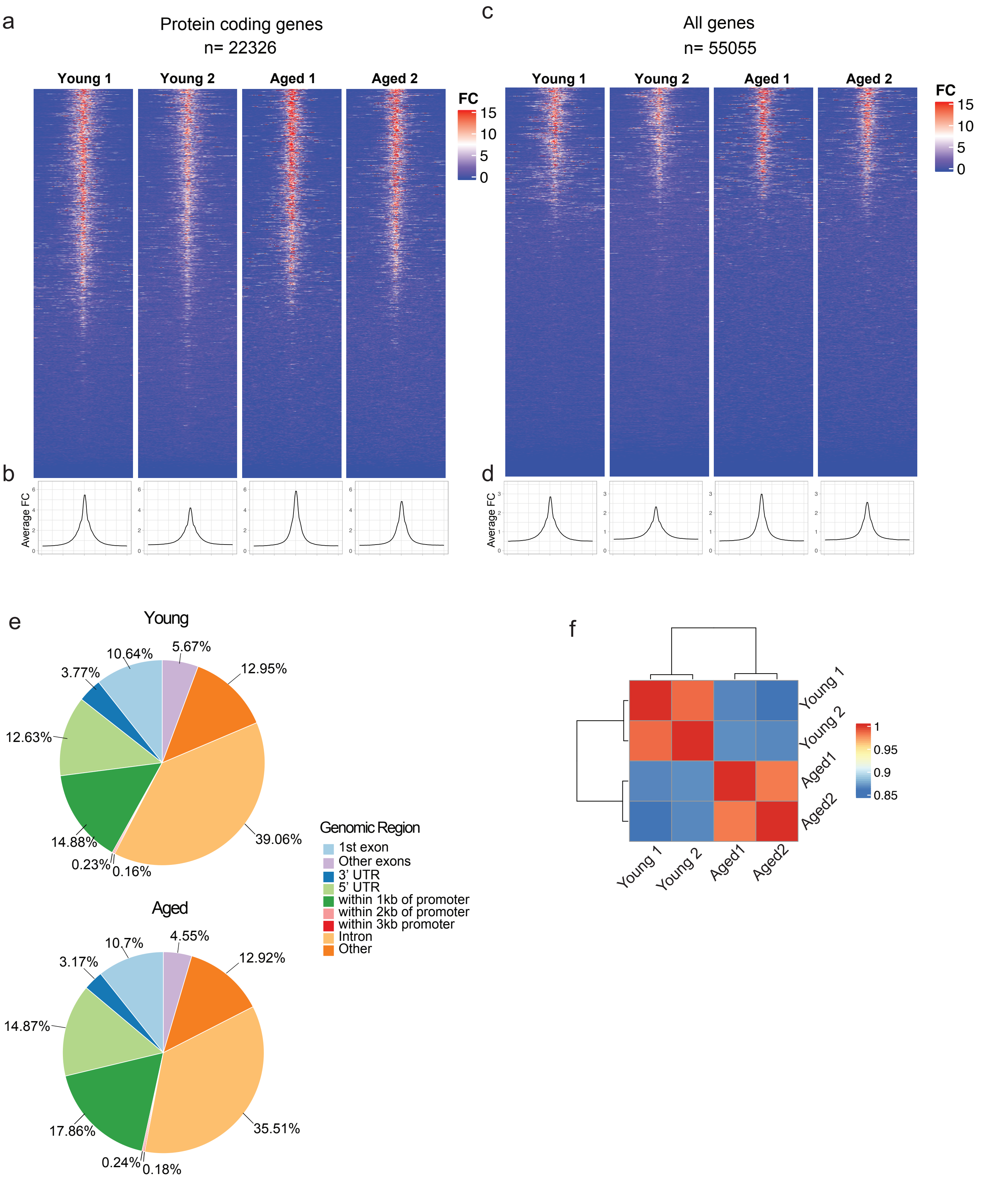

Supplementary Fig. 13: Analysis of chromatin state by ATAC-seq from young and aged MuSCs

a, Pileup of ATAC-seq reads around the TSS of protein-coding genes in young and aged MuSCs.
b, Aggregation plots (Averageograms) showing the position of peaks around the TSS for protein-coding genes in young and aged MuSCs.
c, Pileup of ATAC-seq reads around the TSS of all genes in young and aged MuSCs.
d, Aggregation plots (Averageograms) showing the position of peaks around the TSS for all genes in young and aged MuSCs.
e, Pie charts showing proportions of ATAC-seq peaks throughout the genome in young and aged MuSC samples (n=2 biological replicates per group).
f, Heat map showing the level of similarity between young and aged MuSC ATAC-seq samples (n=2).

Supplementary Figure 14

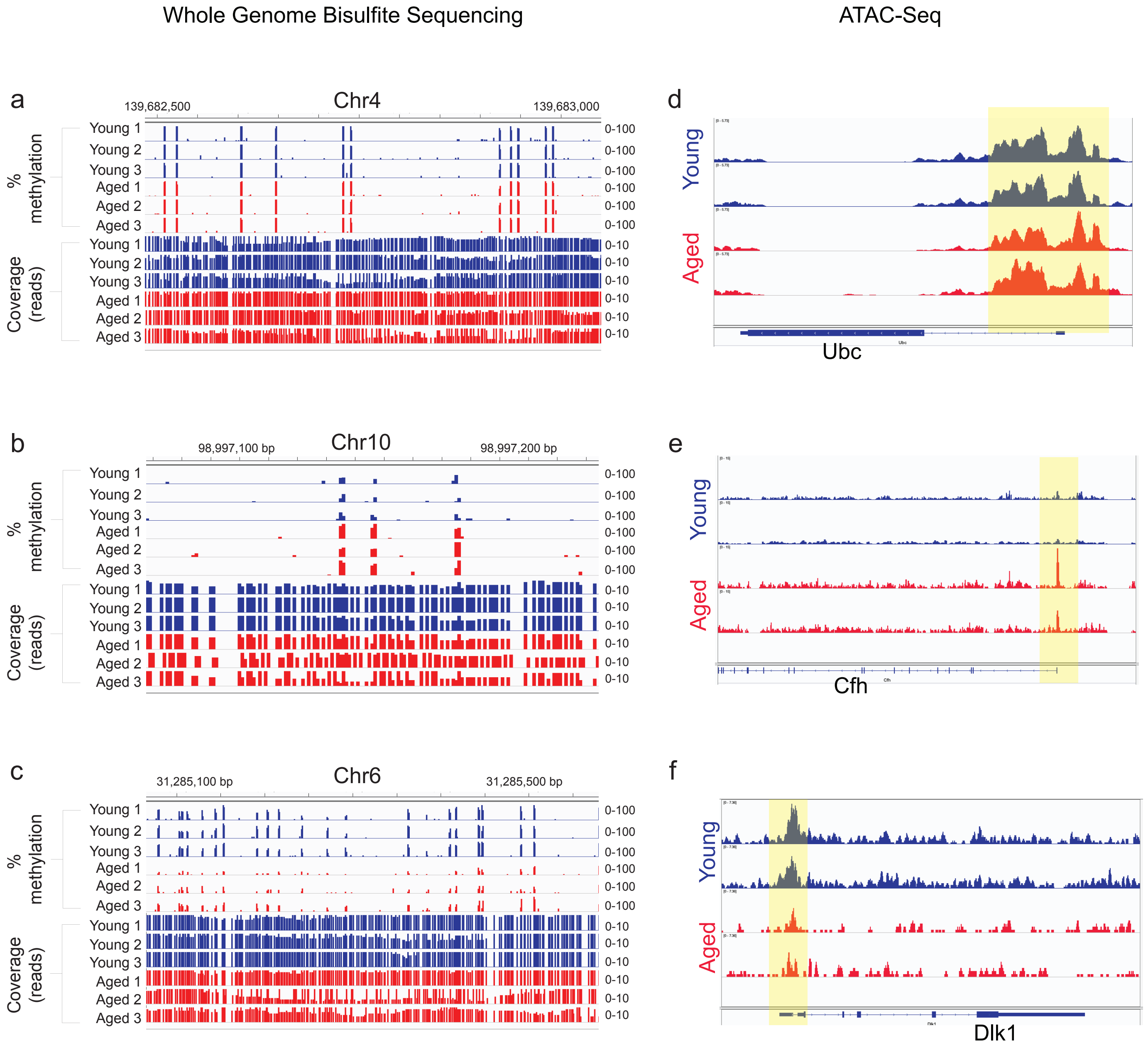

Supplementary Fig. 14: Chromatin is a driver of the age-related changes in MuSCs.
a-c, Representative IGV tracks showing examples of chromosomal regions of equal DNA methylation (a), increased methylation (b), and decreased methylation (c), in aged compared to young MuSCs.
d-f, Representative IGV tracks of ATAC-seq reads for select example genes in young and aged MuSCs showing equal chromatin accessibility (d), increased accessibility (e), and decreased accessibility (f), in aging. Yellow boxes indicate peaks close to the TSS of each gene.

Supplementary Figure 15

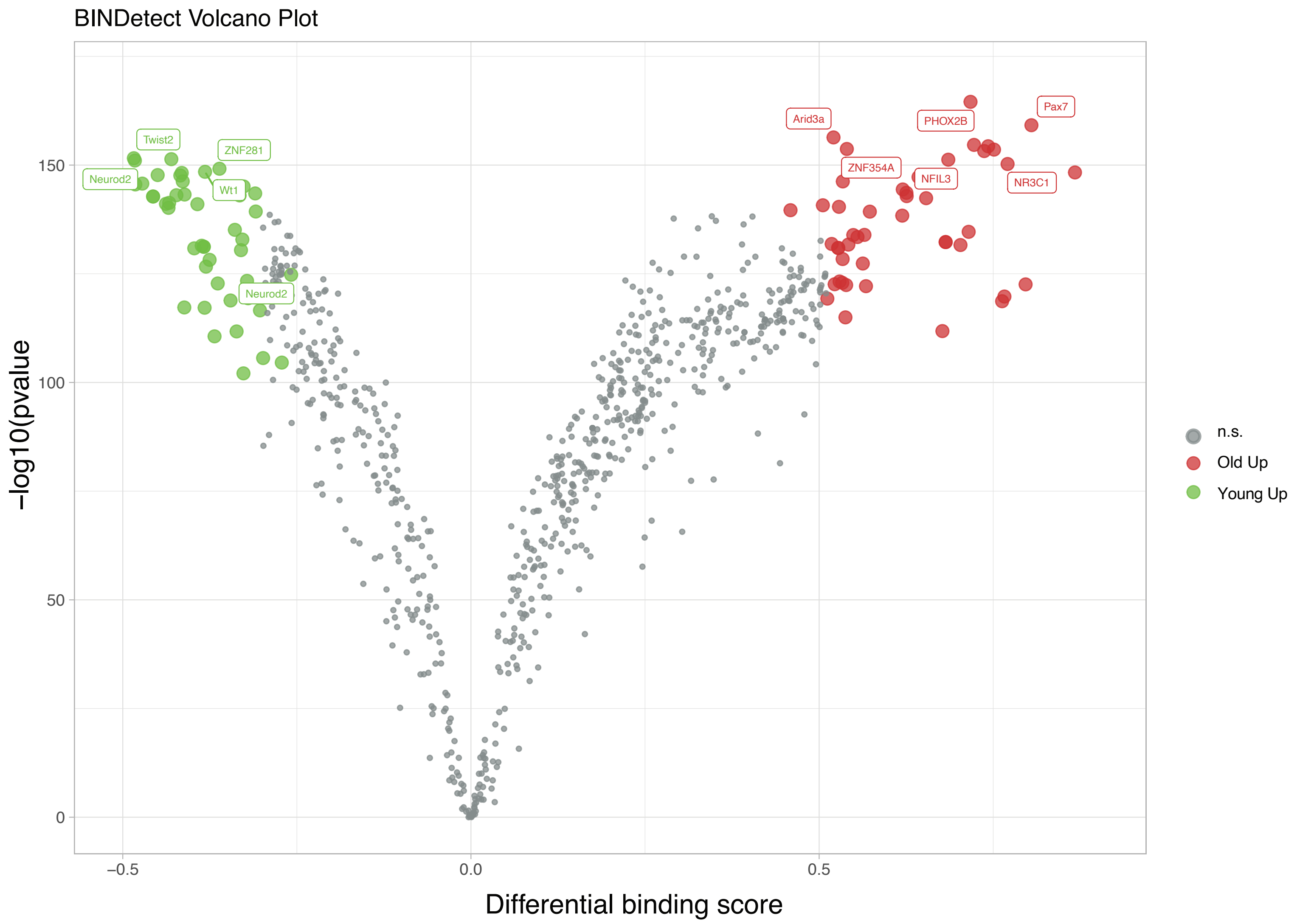

Supplementary Fig. 15: Transcription Factor foot printing between young and aged MuSCs reveals differentially accessible TF binding sites.

Volcano plot showing transcription factor foot printing using TOBIAS[10] of differentially accessible sites between young an aged MuSCs.

References

1 Pasut, A., Oleynik, P. & Rudnicki, M. A. Isolation of muscle stem cells by fluorescence activated cell sorting cytometry. *Methods Mol Biol* **798**, 53-64, doi:10.1007/978-1-61779-343-1_3 (2012).

2 Farup, J., Madaro, L., Puri, P. L. & Mikkelsen, U. R. Interactions between muscle stem cells, mesenchymal-derived cells and immune cells in muscle homeostasis, regeneration and disease. *Cell Death & Disease* **6**, e1830-e1830, doi:10.1038/cddis.2015.198 (2015).

3 Low, M., Eisner, C. & Rossi, F. in *Muscle Stem Cells: Methods and Protocols* (eds Eusebio Perdiguero & D. D. W. Cornelison) 179-189 (Springer New York, 2017).

4 Schaum, N. *et al.* Single-cell transcriptomics of 20 mouse organs creates a Tabula Muris. *Nature* **562**, 367-372, doi:10.1038/s41586-018-0590-4 (2018).

5 Naba, A. *et al.* The matrisome: in silico definition and in vivo characterization by proteomics of normal and tumor extracellular matrices. *Molecular & cellular proteomics : MCP* **11**, M111.014647, doi:10.1074/mcp.M111.014647 (2012).

6 Liberzon, A. *et al.* The Molecular Signatures Database (MSigDB) hallmark gene set collection. *Cell Syst* **1**, 417-425, doi:10.1016/j.cels.2015.12.004 (2015).

7 Gelain, D. P. *et al.* A systematic review of human antioxidant genes. *Frontiers in bioscience (Landmark edition)* **14**, 4457-4463, doi:10.2741/3541 (2009).

8 Picelli, S. *et al.* Full-length RNA-seq from single cells using Smart-seq2. *Nat Protoc* **9**, 171-181, doi:10.1038/nprot.2014.006 (2014).

9 Adey, A. *et al.* Rapid, low-input, low-bias construction of shotgun fragment libraries by high-density in vitro transposition. *Genome biology* **11**, R119, doi:10.1186/gb-2010-11-12-r119 (2010).

10 Bentsen, M. *et al.* ATAC-seq footprinting unravels kinetics of transcription factor binding during zygotic genome activation. *Nature Communications* **11**, 4267, doi:10.1038/s41467-020-18035-1 (2020).

Source Data (Uncropped Scans)

Supplementary Figure 9c

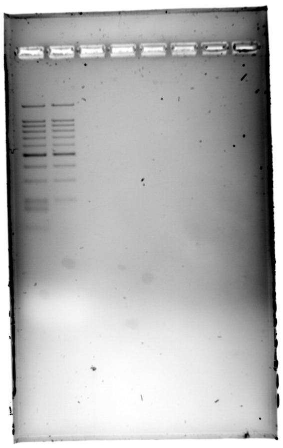

Supplement: Supplementary file 1 — Supplementary Info [file 41467_2023_36265_MOESM1_ESM.pdf]
