# Supplementary material for: Transcriptional reprogramming of skeletal muscle stem cells by the niche environment
Source: Nat Commun. 2023 Feb 1;14:535. doi: 10.1038/s41467-023-36265-x (PMC9892560; doi:10.1038/s41467-023-36265-x)
Supplement: Supplementary file 3 — Description of Additional Supplementary Files [file 41467_2023_36265_MOESM3_ESM.pdf]

Description of Additional Supplementary Files:

Supplementary Data 1: List of the top differentially expressed genes between young and aged MuSCs, FAPs and macrophages (s-value<0.05, LFC>1).

Supplementary Data 2: List of reversible and irreversible genes that are affected by aging (moderated LFC>1, s-value<0.05).

Supplementary Data 3: List of reversible and irreversible genes that are affected by aging (LFC>1, svalue<0.15, LFC batch<0.5).

Supplementary Data 4: List of differentially methylated regions (DMRs) in young compared to aged MuSCs.

Supplementary Data 5: Differentially accessible ATAC-seq peaks between young and aged MuSCs (LFC>1, adjusted p<0.05).
