# Supplementary material for: Transcriptional reprogramming of skeletal muscle stem cells by the niche environment
Source: Nat Commun. 2023 Feb 1;14:535. doi: 10.1038/s41467-023-36265-x (PMC9892560; doi:10.1038/s41467-023-36265-x)
Supplement: Supplementary file 9 — Reporting Summary [file 41467_2023_36265_MOESM9_ESM.pdf]

nature portfolio

Corresponding author(s): Vahab D. Soleimani
Hamed S. Najafabadi

Last updated by author(s): January 12, 2023

Reporting Summary

Nature Portfolio wishes to improve the reproducibility of the work that we publish. This form provides structure for consistency and transparency in reporting. For further information on Nature Portfolio policies, see our Editorial Policies and the Editorial Policy Checklist.

Statistics

For all statistical analyses, confirm that the following items are present in the figure legend, table legend, main text, or Methods section.

n/a	Confirmed	
☐	☒	The exact sample size (*n*) for each experimental group/condition, given as a discrete number and unit of measurement
☐	☒	A statement on whether measurements were taken from distinct samples or whether the same sample was measured repeatedly
☐	☒	The statistical test(s) used AND whether they are one- or two-sided<br>*Only common tests should be described solely by name; describe more complex techniques in the Methods section.*
☐	☒	A description of all covariates tested
☐	☒	A description of any assumptions or corrections, such as tests of normality and adjustment for multiple comparisons
☐	☒	A full description of the statistical parameters including central tendency (e.g. means) or other basic estimates (e.g. regression coefficient) AND variation (e.g. standard deviation) or associated estimates of uncertainty (e.g. confidence intervals)
☐	☒	For null hypothesis testing, the test statistic (e.g. *F*, *t*, *r*) with confidence intervals, effect sizes, degrees of freedom and *P* value noted<br>*Give P values as exact values whenever suitable.*
☒	☐	For Bayesian analysis, information on the choice of priors and Markov chain Monte Carlo settings
☐	☒	For hierarchical and complex designs, identification of the appropriate level for tests and full reporting of outcomes
☐	☒	Estimates of effect sizes (e.g. Cohen's *d*, Pearson's *r*), indicating how they were calculated

Our web collection on statistics for biologists contains articles on many of the points above.

Software and code

Policy information about availability of computer code

Data collection	Sequencing data was obtained using an Illumina NextSeq500 sequencing machine.
Data analysis	All Next-generation sequencing data was analyzed using the software below:<br>R version 4.0.0<br>DESeq2_1.28.1<br>tidyverse_1.3.0<br>cutadapt version 3.4<br>bismark version 0.22.2<br>DMRcaller version 1.6.0.1<br>Diffrac 9bda6c4533d45e3f6da39fa428c659009ff21126 (github commit)<br>GREAT version 4.0.4<br>MACS2 version 2.2.6<br>TOBIAS version 0.12.10<br>Graphpad Prism version 8

For manuscripts utilizing custom algorithms or software that are central to the research but not yet described in published literature, software must be made available to editors and reviewers. We strongly encourage code deposition in a community repository (e.g. GitHub). See the Nature Portfolio guidelines for submitting code & software for further information.

Data

Policy information about availability of data

All manuscripts must include a data availability statement. This statement should provide the following information, where applicable:

- Accession codes, unique identifiers, or web links for publicly available datasets
- A description of any restrictions on data availability
- For clinical datasets or third party data, please ensure that the statement adheres to our policy

The Next-Generation Sequencing (NGS) data reported in this manuscript are available through

NCBI's Gene Expression Omnibus database under super series GSE171998. Individual

accession numbers for WGBS, scRNA-seq, RNA-seq, and ATAC-seq are GSE171604,

GSE171794, GSE171997, and GSE171534, respectively.

Human research participants

Policy information about studies involving human research participants and Sex and Gender in Research.

Reporting on sex and gender	N/A
Population characteristics	N/A
Recruitment	N/A
Ethics oversight	N/A

Note that full information on the approval of the study protocol must also be provided in the manuscript.

Field-specific reporting

Please select the one below that is the best fit for your research. If you are not sure, read the appropriate sections before making your selection.

[x] Life sciences   [ ] Behavioural & social sciences   [ ] Ecological, evolutionary & environmental sciences

For a reference copy of the document with all sections, see nature.com/documents/nr-reporting-summary-flat.pdf

Life sciences study design

All studies must disclose on these points even when the disclosure is negative.

Sample size	For both bulk and single-cell RNA-Sequencing experiments, we included at minimum n=3 biological replicates per condition. For ATAC-Seq, we have included n=2 young and aged samples. No statistical method was used to determine sample size. Sample size was chosen based on convention in the field. For transplantation RNA-Seq data, we initially ran n=3 samples per group. Due to the heterogeneity that occurs in aging, and particularly in aged transplanted samples, we later decided to perform additional transplantation experiments for a total of at least n=5 per group.
Data exclusions	For quality control, we have excluded any transplantation RNA-Seq samples that showed signs of inclusion of contaminating non-muscle cells. We have maintained at least n=3 for all RNA-Seq conditions, even after these exclusions.
Replication	We have performed bulk transplantation RNA-Seq on two occasions, in 2 separate batches that were sequenced separately. Replication was successful.<br>For single-cell RNA-Seq, libraries from each young and aged mouse were created independently as opposed to pooling before library preparation. MuSCs, FAPs and macrophages were isolated by FACS and encapsulated for scRNA-Seq on three separate occasions (at least 1 aged and 1 mouse processed each time). All scRNA-Seq libraries were sequenced in a single sequencing run.
Randomization	Mice were allocated into experimental groups based on their age, since we compare young vs aged mice.Mice were allocated randomly in all experiments.
Blinding	Blinding was not possible during isolation of muscle stem cells from young and aged mice due to visible differences between the two. However, all subsequent analysis was unbiased, since all samples regardless of age were inputed into the same analysis pipeline.

Reporting for specific materials, systems and methods

We require information from authors about some types of materials, experimental systems and methods used in many studies. Here, indicate whether each material, system or method listed is relevant to your study. If you are not sure if a list item applies to your research, read the appropriate section before selecting a response.

Materials & experimental systems

n/a	Involved in the study	
☐	☒	Antibodies
☐	☒	Eukaryotic cell lines
☒	☐	Palaeontology and archaeology
☐	☒	Animals and other organisms
☒	☐	Clinical data
☒	☐	Dual use research of concern

Methods

n/a	Involved in the study	
☒	☐	ChIP-seq
☐	☒	Flow cytometry
☒	☐	MRI-based neuroimaging

Antibodies

Antibodies used	Antibodies used are below:<br>Anti-mouse ITGA7-647 R&D systems FAB3518R (1:500 dilution)<br>Anti-mouse SCA1 (LY6A/E)-FITC BD Pharmigen 557405 (1:5000 dilution)<br>Anti-mouse CD31-PE Invitrogen 12-0311-81 (1:5000 dilution)<br>Anti-mouse CD45-BV785 Biolegend 103149 (1:5000 dilution)<br>Anti-mouse F4/80-APC-Cy7 Biolegend 123117 (1:1000 dilution)<br>Anti-mouse CD11b-PerCP-Cy5.5 Biolegend 101227 (1:5000 dilution)<br>PDGFRA rabbit mAb Cell Signaling 3174 (1:1000 dilution)<br>Anti-mouse F4/80 Invitrogen 12-4801-82 (1:000 dilution)<br>Anti-Mouse Pax7 DSHB AB_528428 (1:100 dilution)<br>Laminin Sigma L9393 (1:750 dilution)<br>GFP invitrogen A-11120 (1:500 dilution)
Validation	All antibodies used have been validated in the published literature (Pasut A. Methods Molecular Bio. 2012; X Feng, J Vis Exp, 2018; Sincennes MC, Nature Comm, 2021; Farup J, Cell Death & Disease, 2015; Low M, Muscle Stem Cells: Methods and protocols, 2017, and more)<br><br>Furthermore, all FACS and fluorescence antibodies have been validated using appropriate negative controls (unstained controls or controls using secondary antibody only). Finally, certain antibodies were validated by demonstrating signal by two different, independent methods (flow cytometry and immunostaining).

Eukaryotic cell lines

Policy information about cell lines and Sex and Gender in Research

Cell line source(s)	primary myoblasts, derived from FACS-sorted muscle stem cells from young and aged C57Bl6 mice
Authentication	These cell lines are authenticated by their expression of ITGA7 and PAX7, and their ability to differentiate into myotubes
Mycoplasma contamination	Negative
Commonly misidentified lines<br>(See ICLAC register)	N/A

Animals and other research organisms

Policy information about studies involving animals; ARRIVE guidelines recommended for reporting animal research, and Sex and Gender in Research

Laboratory animals	C57BL/6J, Tg(Pax7-EGFP), NOD-Prkdcem26Cd52Il2rgem26Cd22/NjuCrl (NCG) mice. Young mice are 5 weeks-old, aged mice are between 22 and 26 months old. Mice were housed at a temperature of 21° and 20% humidity, with a light/dark cycle of 14 hours light/10 hours dark
Wild animals	the study did not involve wild animals

Reporting on sex	All experiments were performed on male mice, to minimize RNA-Seq variation.
Field-collected samples	Study did not involve field-collected samples
Ethics oversight	Animal protocols were approved by the McGill University Animal Care Committee (UACC)

Note that full information on the approval of the study protocol must also be provided in the manuscript.

Flow Cytometry

Plots

Confirm that:

- [x] The axis labels state the marker and fluorochrome used (e.g. CD4-FITC).
- [x] The axis scales are clearly visible. Include numbers along axes only for bottom left plot of group (a 'group' is an analysis of identical markers).
- [x] All plots are contour plots with outliers or pseudocolor plots.
- [x] A numerical value for number of cells or percentage (with statistics) is provided.

Methodology

Sample preparation	Briefly, hindlimb muscle samples were dissected from mice and digested in a Collagenase D/Dispase II/CaCl2 solution. Cells were filtered, resuspended in 2%FBS/PBS and stained with appropriate antibodies. Cells were washed, centrifuged, and resuspended in 2%FBS/PBS for flow cytometry
Instrument	Cells were sorted using a BD FACSAria Fusion flow cytometer.
Software	flow cytometry data was collected using BD FACSDiva software. Figures were subsequently created using FlowJo software
Cell population abundance	FAPs made up ~20-50% of the sorted cells, macrophages made up ~20-35%, and muscle stem cells made up ~40-85% of the cells sorted. These percentages vary based on the age of the mouse, which is shown in Figure 1d. Cell purity was confirmed by immunostaining the 3 sorted cell populations for known markers (PDGFRa, F4/80 and PAX7, respectively), as shown in Supplemental Figure 1c. Purity was found to be above 90% in all cases.
Gating strategy	The gating strategy is present in Figure 1b and Supplemental Figure 1a-b. Briefly, live mononuclear cells were first sorted based on Hoechst expression. Next, endothelial cells were removed by gating CD31- cells. Immune cells were then separated using CD45. CD45+ immune cells were further gated for F4/80+/CD11b+ cells to obtain macrophages. CD45- non-immune cells were further gated using Sca1 and ITGA7. The Sca1+/ITGA7- cell population corresponded to FAPs. The Sca1-/ITGA7+ cell population corresponded to muscle stem cells.

- [x] Tick this box to confirm that a figure exemplifying the gating strategy is provided in the Supplementary Information.
